# Recurrent Novel *P2RY8/IGH* Translocations in B-Lymphoblastic Leukemia/Lymphoma

**DOI:** 10.3389/fonc.2022.896858

**Published:** 2022-07-14

**Authors:** Yanglan Fang, Man Wang, Shuhong Hu, Tanzhen Wang, Yujie Liu, Jinyan Xiao, Yiming Cai, Ying Wang, Huiying Qiu, Xiaowen Tang, Suning Chen, Depei Wu, Yang Xu, Tianhui Liu

**Affiliations:** ^1^ National Clinical Research Center for Hematologic Diseases, Jiangsu Institute of Hematology, The First Affiliated Hospital of Soochow University, Suzhou, China; ^2^ Institute of Blood and Marrow Transplantation, Collaborative Innovation Center of Hematology, Soochow University, Suzhou, China

**Keywords:** translocation, fusion gene, B-lymphoblastic leukemia/lymphoma, *P2RY8*, *IGH*

## Abstract

Translocations involving the immunoglobulin heavy chain (*IGH*) locus are common abnormalities in B-lymphoblastic leukemia/lymphoma (B-ALL) and multiple myeloma. These rearrangements result in a juxtaposition of *IGH* enhancers to the vicinity of oncogenes, such as *MYC* and *CRLF2*, leading to the upregulation of oncogenes. Here, we identified recurrent novel *P2RY8/IGH* translocations in three B-ALL patients by transcriptome sequencing. Noncoding exon 1 of *P2RY8* was translocated to different sites of the *IGH* gene, resulting in transcripts of *P2RY8/IGHM*, *P2RY8/IGHV*, and *P2RY8/IGHD*. However, a high expression level of truncated *P2RY8* was observed in the patients compared with healthy donors, which might be related to the aggressive clinical course and inferior outcome. In summary, we described recurrent novel *P2RY8/IGH* translocations with high expression levels of *P2RY8*, which may contribute to the guidelines for clinical diagnosis and treatment.

## Introduction

B-lymphoblastic leukemia/lymphoma (B-ALL) is a malignant disorder that originates from precursor B cells. The genetic basis of B-ALL is characterized by chromosomal rearrangement, structural variations, and sequence mutations that perturb lymphocyte development, growth, and epigenetic regulation. The genetic abnormalities that drive disease progression impact prognosis and treatment stratification (1, 2).

Immunoglobulin heavy chain *(IGH*) translocations are common and early oncogenic events in B cells and plasma cell malignancies (3). It has been reported that the recurrent translocation partner genes of *IGH* in B-ALL include five members of the CCAAT/enhancer binding protein (CEBP) family of transcription factors (4), the cytokine receptor for erythropoietin (*EPOR*) (5), the type I cytokine receptor *CRLF2* (6) and *IL3* (7). In most cases, translocations involving *IGH* result in the transcriptional activation of its partner genes (8). For example, the *IGH/CRLF2* translocation produces an overexpression of the *CRLF2* protein, resulting in the constitutive activation of the JAK/SAT signaling pathways in leukemic blast cells (9).

In this study, we found new *IGH* rearrangements in three B-ALL patients with recurrent novel *P2RY8/IGH* translocations. The noncoding exon 1 of the purinergic receptor P2Y, G protein coupled, 8 (*P2RY8*) gene was translocated with different sites of *IGH*, resulting in *P2RY8/IGHM*, *P2RY8/IGHV*, and *P2RY8/IGHD* transcripts. A high expression level of *P2RY8* was found in two patients with an aggressive clinical course and poor prognosis, indicating that high levels of *P2RY8* may contribute to leukemogenesis or disease progression.

## Materials and Methods

### Fluorescence *In Situ* Hybridization

FISH analyses were performed according to our institutional protocols (10). Accordingly, a commercial panel of FISH probes covering Philadelphia chromosome-like B-lymphoblastic leukemia (Ph-like ALL), including *ABL1*, *ABL2*, *CRLF2*, *EPOR*, and *JAK2*, was purchased (Vysis, Abbott/IL/USA). A positive rearrangement was reported when at least 3% of the nuclei showed break-apart split signals. Bone marrow (BM) samples at diagnosis or relapse were analyzed by FISH (Olympus IX 71, Tokyo, Japan).

### RNA Sequencing and Reverse Transcription-Polymerase Chain Reaction (RT–PCR)

Total RNA was extracted from the BM samples taken at the time of diagnosis or relapse using an RNeasy Mini Kit (QIAgen, Hilden, Germany). RNA sequencing libraries were prepared using 20-100 ng total RNA of BM samples with the TruSeq RNA library preparation kit v2 (Illumina, CA, USA). Paired-end sequencing with a read length of 150 bp was performed on Illumina NovaSeq platforms to at least 12 G raw data per sample according to the manufacturer’s protocol. The prediction of fusion genes was analyzed by STAR-fusion software (version: 1.9.0) and Fusion catcher (version:1.33) and aligned to the human reference genome GRCh38 (hg38). SNVs/indels were analyzed by following the GATK best practices for variant calling on RNA sequencing. The forward and reverse primer sequences used for the detection of the *P2RY8/IGH* translocations by reverse transcription-polymerase chain reaction (RT–PCR) were as follows: Patient 1 (forward: 5′-CTT AAG CGT TGC ATC CTG TT-3′, reverse: 5′-GCT GTT ATC CTT TGG GTG TCT-3′), Patient 2 (forward: 5′-CTG GAC AGA TGG AAC TGG AAG G-3′, reverse: 5′- ATA AGC AGT GGA TGT GTG TGG-3′) and Patient 3 (forward: 5′-AAG GTT GCT GGA CAG ATG GAA C-3′, reverse: 5′-TTT CTT TGT TGC CGT TGG GGT-3′).

### Real-Time Quantitative Polymerase Chain Reaction (RT–qPCR)

cDNA synthesis was performed by the Reverse Transcription Reagent Kit (Applied Biological Materials Inc., BC, Canada) according to the manufacturer’s instructions. RT–qPCR was performed using TB Green Premix Ex Taq II (Takara Bio, Otsu, Japan) according to the manufacturer’s instructions. All experiments were performed in triplicate with an ABI QuantStudio 3 Real-Time PCR System (Applied Biosystems, MA, USA). Differences were calculated by the ΔΔCt comparative quantization method using *GAPDH* as an internal control. The specific primers of *P2RY8* used for RT–qPCR were as follows: P1 (forward: 5′-TTC CTC TTC ACC ATC TTC ATC CTG-3′, reverse: 5′-CGT GGT AGT AGC TCT TGC CGT AGA-3′) and P2 (forward: 5′-CCT TTG CAA GGT TGC TGG AC-3′, reverse: 5′- TGT TTG CGT AAA AGG CCA CG-3′).

## Results

**Figure 1:**
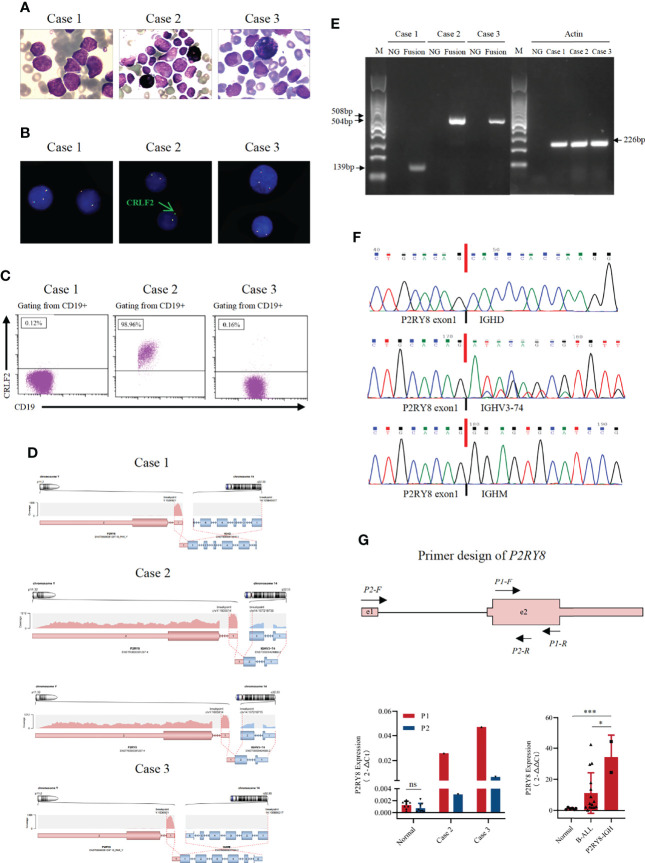
Identification of novel recurrent P2RY8/IGH fusions. **(A)** Bone marrow aspirate at diagnosis or relapse (Wright’s staining ×1,000). **(B)** Fluorescence *in situ* hybridization results with a *CRLF2* break-apart probe, which detected split signals of *CRLF2*. **(C)** Immunophenotyping of bone marrow cells at diagnosis or relapse. Flow cytometric analyses of leukemic cells revealed that the blast cells in Case 2 were positive for *CRLF2*. **(D)** RNA sequencing analysis revealed one breakpoint in exon 1 of the *P2RY8* gene and breakpoints in different exons of the *IGH* gene in three patients. **(E)** A 139 bp product for Case 1 and a 508 bp product for Case 2 were detected by RT–PCR in samples taken at diagnosis. A product of 504 bp for Case 3 was detected by RT–PCR in samples taken at relapse. **(F)** Sequence alignment of the amplified product revealed breakpoints between exon 1 of the *P2RY8* gene and different exons of the *IGH* gene at diagnosis or relapse. **(G)** Structure of the primers designed for the RT–qPCR of *P2RY8*. The amount of *P2RY8* mRNA in BMNC cell fractions purified from *P2RY8-IGH* patients, healthy volunteers (normal) and B-ALL patients was determined by real-time RT–PCR analysis. Each fold change was calculated by the 2^(-△Ct) method (left) and 2^(-△△Ct) method (right). Values are the mean ± SEM. *p < 0.05, **p < 0.01, ***p < 0.001.

In Case 1, a 17-year-old female was admitted due to fatigue and worsening asthenia. Her blood tests showed a white blood count of 1.56×10^9^/L, hemoglobin 84 g/L, and platelets 54×10^9^/L. Bone marrow (BM) smears showed 75% blasts (**Figure 1A**). The blasts were positive for CD34, HLA-DR, CD10, CD19, and CD123 and partially positive for CD22 (**Table 1**). Conventional karyotyping was normal. The results of multiplex RT–PCR covering 43 acute leukemia-related fusion genes were negative. FISH analysis with split signal probes covering *ABL1*, *JAK2*, *CRLF2*, and *EPOR* all tested negative (**Figure 1B**). Next-generation sequencing (NGS) targeting 172 leukemia- and lymphoma-related genes identified *ANKRD26-p.Asn267Ser*, *PTPN11-p.Glu76Lys*, *CIITA-p.His1119Asn* and *FAT1-p.Phe765Tyr* mutations. The patient was treated with conventional induction therapy (IVP regimen, containing idarubicin, vincristine, and dexamethasone) and achieved complete remission. Subsequently, she received consolidation therapy with cyclophosphamide, 6-MP, and arabinoside cytosine for one cycle and high-dose methotrexate (MTX) for one cycle. Then, she received anti-CD19 chimeric antigen receptor-modified T-cell therapy bridging to haploidentical allogeneic hematopoietic stem cell transplantation (allo-HSCT) and remained in complete remission until the last follow-up in October 2021.

**Table 1:** Patient characteristics and clinical outcome.

Case	Age/Gender	Time	Blast(%)	Phenotype			Karyotype	Fusiongene	Mutation	Treatment	Clinical outcome
				CD19	CD22	CRLF2					
1	17/F	Initial diagnosis	75	+	+	–	Normal	*P2RY8/IGHD*	*ANKRD26-p.Asn267Ser* *PTPN11-p.Glu76Lys* *CIITA-p.His1119Asn* *FAT1-p.Phe765Tyr*	IVP→*CR*→consolidation chemotherapy→CD19-CAR T→allo-HSCT	Alive
2	22/M	Initial diagnosis	81.5	+	+	+	Normal	*P2RY8/IGHV*	*CIITA-p.Asp206Asn* *SMC2-p.Asp655Val* *TET1-p.Ser1845Pro* *TNFAIP3-p.Ala648Val*	CIVP+Dasatinib→*CR*→ consolidation chemotherapy→relapse→CD19/CD22-CAR T→*NR*→allo-HSCT	Alive
3	40/F	Initial diagnosis	95	+	+	N/A	46, XX, del (9) (p22) [10]/46, XX [10]	Normal	N/A	treatment in local hospital→relapse→VILP→*NR*→IA→*NR*→Blinatumomab→*NR*→local hospital	Dead
		Relapse	90	+	+	–	47, XX, +X,9p- [9]/46, XX [1]	*P2RY8/IGHM*	*NRAS-p.Gly12Asp* *PTPN11-p.Asp61Val* *PTPN11-p.Thr468Met* *KRAS-p.Gly12Val* *CALR-p.Gly108Arg*		

Allo-HSCT, allogeneic hematopoietic stem cell transplantation; CAR T, chimeric antigen receptor T-cell therapy; CIVP, Cyclophosphamide, Idarubicin, Vincristine, Dexamethasone; CR, complete remission; F, female; IA, Idarubicin, Cytarabine;IVP, Idarubicin, Vincristine, Dexamethasone; M, male; N/A, not available; NR, non remission; PR, partial remission; VILP, Vincristine, Idarubicin, L-asparaginase, Dexamethasone.

In Case 2, a 22-year-old male presented with fever, stomach ache, and dizziness. Blood tests showed a white blood count of 24.22×10^9^/L, hemoglobin 90 g/L, and platelets 93×10^9^/L. BM morphology analysis detected 87.5% blasts (**Figure 1A**). The blasts were positive for CD34, CD10, CD19, CD25, CD123, and HLA-DR and partially positive for CD33, CD38, and CD20 (**Table 1**). Cytogenetic analysis showed a normal karyotype. *IGH/CRLF2* rearrangement was detected by FISH and RNA sequencing (**Figure 1B** and **Supplementary Figure 1A**). High expression of *CRLF2* was detected by RNA sequencing (**Supplementary Figure 1B**) and flow cytometry (**Figure 1C**). The results of multiplex RT–PCR were negative. NGS (172 genes) revealed *CIITA-p.Asp206Asn*, *SMC2-p.Asp655Val*, *TET1-p.Ser1845Pro*, and *TNFAIP3-p.Ala648Val* mutations. The patient received conventional chemotherapy, and dasatinib (100 mg, once daily) was administered. Relapse occurred after two cycles of consolidation therapy. Subsequently, he was enrolled in the CAR T-cell clinical trial (NCT03614858). The VP+FC regimen (containing vincristine, dexamethasone, fludarabine, and cyclophosphamide) was administered to reduce the tumor burden. Then, 1.0×10^7^/L tandem CD19/CD22 CAR T cells were infused *via* a dose-escalation schedule for three consecutive days. BM smears showed no response after infusion. The patient received salvaged allo-HSCT in January 2021. He remained in complete remission until the last follow-up in August 2021.

Case 3 involved a 40-year-old female who was a patient with refractory and relapsed B-ALL. At the initial consultation in January 2018, her blood tests showed a white blood count of 3.62×10^9^/L, hemoglobin 59 g/L, and platelets 63×10^9^/L. BM morphology analysis detected 95% blasts, which were positive for CD19, CD34, cCD22, and HLA-DR (**Table 1**). BM karyotyping showed 46, XX, del (9) (p22) [10]/46, XX [10]. She achieved complete remission after receiving conventional induction chemotherapy and then received three courses of consolidation therapy. The treatment was not administered continuously, and the patient was admitted to the local hospital for recurrence in October 2018. A screen for 33 fusion genes associated with Ph-like ALL was negative. The BM smear showed no remission after two courses of induction chemotherapy. The patient was admitted to our hospital in March 2019. BM morphology analysis detected 90% blasts (**Figure 1A**). The blasts were positive for CD34, HLA-DR, CD10, CD19, CD13, CD38, CD79a, and CLL1 and partially positive for CD22. BM karyotyping showed 47, XX, +X,9p- [9]/46, XX [1]. No fusion gene was identified by multiplex RT–PCR. NGS covering 51 genes related to hematological malignancies revealed *NRAS-p.Gly12Asp, PTPN11-p.Asp61Val, PTPN11-p.Thr468Met, KRAS-p.Gly12Val*, and *CALR-p.Gly108Arg* mutations. She underwent induction chemotherapy with the IVP regimen in our hospital, but her BM smear showed no response. Subsequently, she was enrolled in the Blinatumomab clinical trial (CTR20170176) for relapsed/refractory (R/R) ALL. Because of severe cytokine release syndrome, she withdrew from the clinical trial, and blinatumomab was administered for two days (9 µg, d1-d2). A total of 99.8% blast cells were detected in bone marrow aspirates by flow cytometry. She refused further therapy and died of progression in May 2019.

RNA sequencing of bone marrow cells from these three patients was performed, and *P2RY8/IGH* translocations (**Figure 1D**) were identified. RT–PCR and Sanger sequencing further confirmed three different *P2RY8/IGH* transcripts (**Figures 1E, F**). In the three patients, exon 1 of the *P2RY8* gene was fused with the *IGH* gene. However, the fused *IGH* fragments were different. Case 1 and Case 3 have unique transcripts, while Case 2 has two different transcripts. In Case 1, exon 1 of *P2RY8* was fused with exon 1 of immunoglobulin heavy constant delta (*IGHD*). RT–PCR and Sanger sequencing showed a sharp band of 139 bp, which is consistent with *P2RY8/IGHD* fusion. In Case 2, exon 1 of *P2RY8* was fused with exon 2 of immunoglobulin heavy variable 3-74 (*IGHV3-74*) with 2 different fusion sites, resulting in *IGHV3-74/P2RY8* fusion. The two *IGHV3-74* fragments fused with *P2RY8* differed by 15 bases in length. In Case 3, exon 1 of *P2RY8* was fused with exon 1 of immunoglobulin heavy constant mu (*IGHM*). A sharp band of 504 bp was observed, which is consistent with *P2RY8/IGHM* fusion.

*IGH* translocations always lead to the overexpression of partner genes. To determine whether *P2RY8* transcription was influenced by the *P2RY8/IGH* translocation, two pairs of primers were designed (**Figure 1G**). P1 covered *P2RY8* exon 2, and the cDNA of both unbroken and truncated *P2RY8* could be detected. P2 spanned *P2RY8* exon 1, intron 1, and exon 2, representing the unbroken mRNA of *P2RY8*. There was no significant difference in the abundance of *P2RY8* mRNA from healthy donors by quantitative RT–PCR (RT-qPCR) using P1 and P2. However, RT-qPCR results from Case 2 and Case 3 showed that the products of P1 were remarkably higher than those of P2. These findings indicated that truncated *P2RY8*, the derivative of the *P2RY8/IGH* translocation, may be transcribed with an unknown promoter or enhancer. We tried to identify the truncated *P2RY8* transcripts that contain some intron 1 sequence as a suggestion of cryptic promoter activation by *IGH*. RT-PCR was performed in cDNA and genomic DNA from case 2 and 3 by P0 primers, which spanned the junction of exon 2 and intron 1 of *P2RY8* (**Supplementary Figure 2A**). As a result, a sharp band of 846bp, which is consistent with *P2RY8*, was found in the product of genomic PCR. However, no consistent bands were found in the products by cDNA samples (**Supplementary Figures 2B, C**). One possible reason for this failure is that the transcript start sites may be further downstream of the intron primer.

We further detected *P2RY8* mRNA expression in BM MNCs using primer P1 in Case 2 and Case 3 compared with that in healthy donors and B-ALL patients. The results suggested that the expression level of *P2RY8* was increased in *P2RY8/IGH* patients compared with healthy donors (p=0.0004) and B-ALL patients (p=0.03), indicating that high levels of *P2RY8* may contribute to leukemogenesis or disease progression.

## Discussion

The *IGH* gene plays an important role in B-cell development. To produce antibody diversity and enhance the affinity of immunoglobulins to recognize and bind to foreign antigens, DNA undergoes a series of genetic events, including V(D)J rearrangement, somatic hypermutation, and class switch recombination (11–13). This system results in a diversity of high-affinity antibodies but also leaves B cells vulnerable to potentially oncogenic translocations. It has been reported that more than 40 genes can be fused with *IGH*. The common genes are of the *CRLF2*, *ID4*, *EPOR*, *IL3*, and CEBP gene families, which are seen in ALL. Recently, two *IGH* rearrangements were found in myeloid tumors, including an *IGH-MECOM* in myelodysplastic syndrome (MDS) and an *IGH-CCNG1* in acute myeloid leukemia (14). *IGH* translocation juxtaposes the promotors of oncogenes with transcriptional enhancers within the *IGH* locus, resulting in the transcriptional activation of oncogenes (3).

The *P2RY8* gene is located on both chromosomes X and Y. It belongs to the P2Y family of G-protein coupled receptors that mediate migration inhibition and the growth regulation of B cells in lymphoid tissues. *P2RY8* was reported to have oncogenic potential. Loss of function somatic mutations were reported in GC-derived diffuse large B-cell lymphoma (DLBCL) and Burkitt lymphoma (BL) (15–17). *P2RY8* expression was always increased in leukemia patients, especially in those with refractory disease. A high level of *P2RY8* expression was associated with a poorer prognosis than a lower level (18, 19). Thus, overexpression of *P2RY8* may contribute to leukemogenesis or progression.

Here, we found recurrent novel *P2RY8/IGH* translocations in three B-ALL samples by transcriptome sequencing. Although we confirmed the existence of three transcripts by RT-PCR, we failed to observe the translocations by whole genome sequencing. It seems that these *P2RY8/IGH* transcripts might be special products in the RNA splicing process. The same noncoding exon 1 of *P2RY8* fused with different sites of the *IGH* gene, resulting in *P2RY8/IGHM*, *P2RY8/IGHV*, and *P2RY8/IGHD* transcripts. *P2RY8* may use the exon1/intron 1 splice junction while *IGH* may have different splicing sites. However, these results do not completely exclude the possibility of complex genomic events occurring in a small proportion of the tumor cells and generating the RNA chimera.

It has been reported that *IGH* translocations always result in high expression of partner genes by juxtaposing the promotors of the partner gene with transcriptional enhancers within the *IGH* locus. We then designed the two pairs of primers to distinguish the full length of the *P2RY8* transcript and the truncated *P2RY8* transcript (coding region). Interestingly, in these three cases, truncated *P2RY8* transcript was examined with high expression, while full-length of *P2RY8* transcript was detected at a low level. Moreover, the *P2RY8* mRNA expression level was also higher than that in healthy donors and B-ALL patients. Thus, we speculated that the cryptic promoter of *P2RY8* might be activated by *IGH*, resulting in the overexpression of the truncated *P2RY8* transcripts. To explore the existence of a truncated *P2RY8* transcript that contains some intron 1 sequence as a suggestion of cryptic promoter activation by *IGH*, RT-PCR was performed in both cDNA and genomic DNA from case 2 and 3. A sharp band of 846bp was observed in the product using genomic DNA sample other than that using cDNA samples. The results might be due to the fact that the start sites of the truncated transcripts were downstream of the intron primer. In summary, *P2RY8/IGH* transcripts are not capable of producing *P2RY8* protein. These fusion transcripts are simply an indication of the *IGH/P2RY8* translocation and are not pathogenic. We considered that the pathogenicity is medicated by the overexpression of the truncated *P2RY8* transcripts that are capable of generating the *P2RY8* protein.

Another interesting phenomenon was observed in one of three patients: *IGH/CRLF2* transcript and *P2RY8/IGH* transcript were coexistent in case 2, and that was not been reported before. Relapse occurred when he was treated with conventional chemotherapy and tyrosine kinase inhibitor (TKI) therapy. Tandem CD19/CD22 CAR T infusion also didn’t work. Finally, he received salvaged allo-HSCT. The overexpression of *P2RY8* was also observed in this case. It remains a problem if *P2RY8* overexpression here could be a diver fact when coexisting with *IGH/CRLF2*. Meanwhile, there is a possibility that *P2RY8* overexpression might be a second hit in this subtype of B-ALL.

In summary, we described recurrent novel *P2RY8/IGH* translocations with high expression levels of *P2RY8*. Future studies are needed to define the biological significance of this event in leukemogenesis and may provide contributions to the guidelines for clinical diagnosis and treatment.

## Data Availability Statement

The data presented in the study are deposited in the NCBI Sequence Read Archive (SRA) repository, accession number PRJNA816342.

## Ethics Statement

The studies involving human participants were reviewed and approved by Ethics Committee of the First Affiliated Hospital of Soochow University. Written informed consent to participate in this study was provided by the participants’ legal guardian/next of kin. Written informed consent was obtained from the individual(s), and minor(s)’ legal guardian/next of kin, for the publication of any potentially identifiable images or data included in this article.

## Author Contributions

YW, HQ, and XT treated the patients. MW and SC performed the molecular studies and analysis. YF and SH designed the genetic analysis and wrote the manuscript. TW, YL, JX, and YC analyzed the clinical data. YX and TL critically read and revised the manuscript. All authors approved the final version of the manuscript. All authors contributed to the article and approved the submitted version.

## Funding

This work was supported by grants from the Excellent Youth Science Fund of Jiangsu Province (BK20211553), the Natural Science Foundation of China (81700139, 82070187, 81870120, and 82000157), the Key R&D Program of Jiangsu Province (BE2019655), the Translational Research Grant of NCRCH (2021ZKMB01) and the Natural Science Fund of Jiangsu Province (BK20170360, BK20190173).

## Conflict of Interest

The authors declare that the research was conducted in the absence of any commercial or financial relationships that could be construed as a potential conflict of interest.

## Publisher’s Note

All claims expressed in this article are solely those of the authors and do not necessarily represent those of their affiliated organizations, or those of the publisher, the editors and the reviewers. Any product that may be evaluated in this article, or claim that may be made by its manufacturer, is not guaranteed or endorsed by the publisher.

## Supplementary Material

Supplementary Figure 1
**(A)** Circos plot displaying the interconnectivity between *CRLF2* and *IGH*. **(B)**The expression levels of *CRLF2, P2RY8, IL3RA, CSF2RA*, and *ASMTL* were detected in our RNA sequencing. FPKM: fragments per kilobase of transcript sequence per millions base pairs sequenced.

Supplementary Figure 2
**(A)** P0 Primers designed to identify the truncated *P2RY8* transcript with intron 1 sequence. **(B)** Products of RT-PCR using cDNA samples from patients. No consistent bands of *P2RY8* were found. **(C)** Products of RT-PCR using genomic DNA samples from patients. An 846 bp product consistent with *P2RY8* was detected.
