# Supplementary figures and images for: Recurrent Novel P2RY8/IGH Translocations in B-Lymphoblastic Leukemia/Lymphoma
Source: Front Oncol. 2022 Jul 14;12:896858. doi: 10.3389/fonc.2022.896858 (PMC9330356; doi:10.3389/fonc.2022.896858)

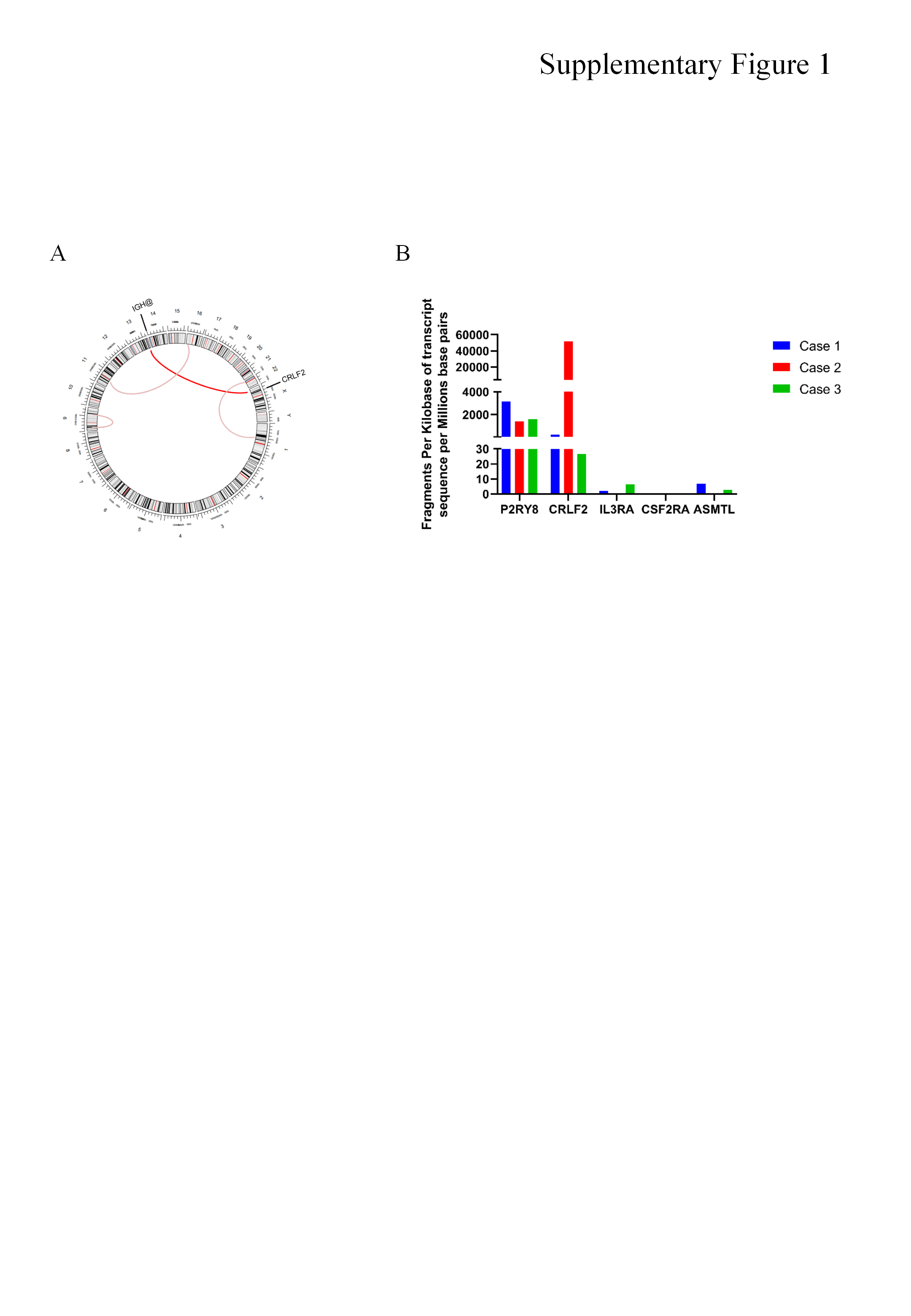

Supplement: Supplementary Figure 1 — (A) Circos plot displaying the interconnectivity between CRLF2 and IGH. (B)The expression levels of CRLF2, P2RY8, IL3RA, CSF2RA, and ASMTL were detected in our RNA sequencing. FPKM: fragments per kilobase of transcript sequence per millions base pairs sequenced. [file Image_1.tif]

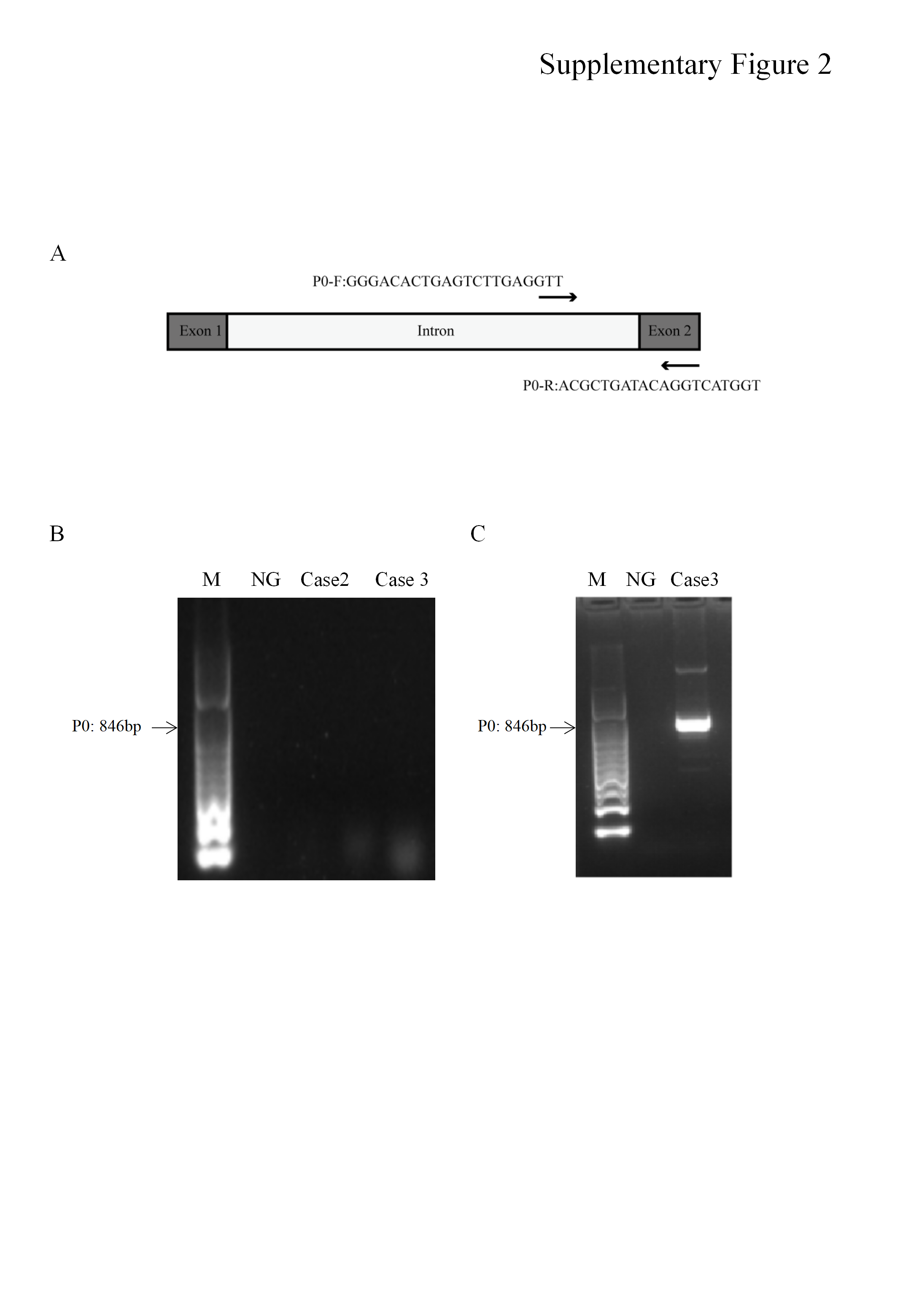

Supplement: Supplementary Figure 2 — (A) P0 Primers designed to identify the truncated P2RY8 transcript with intron 1 sequence. (B) Products of RT-PCR using cDNA samples from patients. No consistent bands of P2RY8 were found. (C) Products of RT-PCR using genomic DNA samples from patients. An 846 bp product consistent with P2RY8 was detected. [file Image_2.tif]
